# Association between diaphragmatic dysfunction after adult cardiovascular surgery and prognosis of mechanical ventilation: a retrospective cohort study

**DOI:** 10.1186/s40560-023-00688-x

**Published:** 2023-09-12

**Authors:** Reimi Inoue, Yusuke Nagamine, Masahide Ohtsuka, Takahisa Goto

**Affiliations:** 1 Intensive Care Department, Yokohama City University Medical Center, 4-57 Urafune, Minami-Ku, Yokohama, Kanagawa 232-0024 Japan; 2 Department of Anesthesiology and Critical Care Medicine, Yokohama City University Hospital, 3-9 Fukuura, Kanazawa-Ku, Yokohama, Kanagawa 236-0004 Japan

**Keywords:** Diaphragmatic dysfunction, Cardiovascular surgery, Mechanical ventilation, Prognosis

## Abstract

**Background:**

Diaphragmatic dysfunction often occurs after adult cardiovascular surgery. The prognostic effect of diaphragmatic dysfunction on ventilatory management in patients after cardiovascular surgery is unknown. This study aimed to investigate the association between diaphragmatic dysfunction and prognosis of ventilatory management in adult postoperative cardiovascular surgery patients.

**Methods:**

This study was a single-center retrospective cohort study conducted at a tertiary care university hospital. This study included adult patients admitted to the intensive care unit under tracheal intubation after cardiovascular surgery. Spontaneous breathing trial was performed, and bilateral diaphragmatic motion was assessed using ultrasonography; diaphragmatic dysfunction was classified as normal, incomplete dysfunction, or complete dysfunction. The primary outcome was weaning off in mechanical ventilation. The duration of mechanical ventilation was defined as duration from the date of ICU admission to the date of weaning off in mechanical ventilation. The secondary outcomes were reintubation, death from all causes, improvement of diaphragm position assessed by chest radiographs. The subdistribution hazard ratio or hazard ratio (HR) with 95% confidence of intervals (CIs) were estimated by Fine-Gray models or Cox proportional hazard models adjusted for potential confounders.

**Results:**

Of 153 patients analyzed, 49 patients (32.0%) had diaphragmatic dysfunction. Diaphragmatic dysfunction consisted of incomplete dysfunction in 38 patients and complete dysfunction in 11 patients. Diaphragmatic dysfunction groups had longer duration of mechanical ventilation (68 h [interquartile range (IQR) 39–114] vs 23 h [15–67], adjusted subdistribution HR 0.63, 95% CIs 0.43–0.92). There was a higher rate of reintubation (12.2% vs 2.9%, univariate logistic regression analysis *p* = 0.034, unadjusted odds ratio = 4.70, 95% CIs 1.12–19.65), and a tendency to have higher death from all causes in the diaphragmatic dysfunction group during follow-up period (maximum 6.5 years) (18.4% vs 9.6%, adjusted HR 1.64, 95% CIs 0.59–4.53). The time to improvement of diaphragm position on chest radiograph was significantly longer in the diaphragmatic dysfunction group (14 days [IQR 6–29] vs 5 days [IQR 2–10], adjusted subdistribution HR 0.54, 95% CIs 0.38–0.77).

**Conclusions:**

Diaphragmatic dysfunction after adult cardiovascular surgery was significantly associated with longer duration of mechanical ventilation and higher reintubation.

## Background

The diaphragm plays an important role in continuous stable spontaneous ventilation. It is innervated by the right and left diaphragmatic nerves derived from the third to fifth cervical spinal nerve roots, and vascularized by the superior, intercostal, and inferior diaphragmatic arteries. Diaphragmatic dysfunction is known to affect mechanical ventilation weaning off.

Especially, after cardiovascular surgery in adults, diaphragmatic dysfunction often occurs due to surgical injury, physical stretch, local hypothermia, and dissection of the internal thoracic artery. The incidence of diaphragmatic dysfunction after cardiovascular surgery in adults is approximately 36% [1, 2]. The prognosis of diaphragmatic dysfunction after cardiac surgery is few reported, and there is no consistent consensus on the prognosis. In uncomplicated elective cardiovascular surgery patients, diaphragmatic function is always impaired in the immediate postoperative period and recovers by the fifth postoperative day [3]. However, few reports exist on the short-term prognostic effect of diaphragmatic dysfunction after cardiovascular surgery on respiratory management, including duration of mechanical ventilation, frequency of reintubation, and mortality. Long-term prognosis, the rate of improvement of diaphragmatic dysfunction, and the time to improvement is not clear.

The aim of this study was to investigate the association between diaphragmatic dysfunction and short- and long-term prognosis of respiratory management in adult postoperative cardiovascular surgery patients.

## Methods

### Design, setting

This study was conducted this single-center retrospective cohort study at a tertiary care university hospital, where approximately 250 adult cardiovascular surgeries are performed annually. Patient data were collected using electronic medical records.

### Participants

We included patients admitted to the intensive care unit (ICU) under tracheal intubation after adult cardiovascular surgery. The use of cardiopulmonary bypass (CPB) during surgery was not distinguished. We excluded patients with a preoperative elevated diaphragm, cases involving a diaphragmatic incision—such as descending aortic or thoracoabdominal arterial replacement, cases of reoperation, and cases in which were difficult in assessing the diaphragm due to non-cooperation with the examination or inability to perform ultrasound examinations. The inclusion period for the patient registry was July 2013 to December 2014.

### Standard protocol of spontaneous breathing trial and extubation criteria at our institution

We performed spontaneous breathing trial (SBT) when patients had no uncontrolled bleeding; controlled pain; good communication; and stable hemodynamics, fluid balance, and electrolytes. Parameters measured were arterial blood gas analysis in spontaneous breathing, minute volume of ventilation, respiratory rate, tidal volume, rapid shallow breathing index (RSBI, respiratory frequency/tidal volume(L)), forced expiratory pressure, and pressure at which the tracheal tube leaks. Patients were assessed for bilateral diaphragmatic motion and pleural effusion using two-dimensional ultrasonography at the SBT routinely.

Extubation criteria were PaO_2_/FiO_2_ > 250 mmHg, PaCO_2_ 35–50 mmHg, respiratory rate < 25 breaths/min, RSBI < 100, minutes volume of ventilation (mL)/preoperative body weight (kg) < 200 mL/kg, tidal volume (mL)/preoperative body weight (kg) > 5 mL/kg, vital capacity (mL)/body weight at preoperative > 10 mL/kg, forced expiratory pressure > 20 cmH_2_O, and pressure at which the tracheal tube cuff leaks < 20 cmH_2_O. If some criteria were not achieved, the final decision to extubate was made by experienced intensivists based on findings of SBT and diaphragmatic dysfunction.

### Exposure

Exposure was defined as the presence of diaphragmatic dysfunction. This study assessed the presence of diaphragmatic dysfunction using ultrasound at the time of the first SBT on bilateral sides. The evaluation was performed with the upper body as upright as possible. Scanning was performed by placing the probe between the ribs, which were not disturbed by drains. As far as possible in B-mode, the head–caudal movement was observed at the apex of the diaphragmatic dome.

Diaphragmatic dysfunction were classified as normal, incomplete dysfunction, or complete dysfunction. Diaphragm movements have been reported to be 2.6–30 mm at rest and 16.7–110 mm during deep breathing [4]. We determined whether diaphragm movement was enough according to the criteria of 20 mm at rest and 100 mm during deep breathing. In accordance with the movement of the diaphragm, we have classified three levels of diaphragmatic movement: normal, incomplete, and complete. Incomplete and complete dysfunction were defined as the diaphragmatic dysfunction group and normal was defined as enough movement toward the caudal side both during rest and deep breathing. Incomplete dysfunction was defined by the movement of the diaphragm to the caudal side on inspiration during rest, without sufficient movement on inspiration during deep breathing. Complete dysfunction was defined by the movement of the diaphragm toward the head side during inspiration at rest (paradoxical movement). Complete dysfunction was also defined as the absence of diaphragmatic movement both during rest and deep breathing. In patients who underwent multiple SBTs, the more severe stage of dysfunction was defined as the final diagnosis.

### Outcome

The primary outcome was weaning off in mechanical ventilation. The duration of mechanical ventilation was defined as duration from the date of ICU admission to the date of weaning off in mechanical ventilation. For patients who used noninvasive positive pressure ventilation (NPPV) after extubation, the date of weaning off in NPPV was defined as the date of weaning off in mechanical ventilation. For patients who were reintubated, the date of weaning off in mechanical ventilation was defined as the date of weaning off in mechanical ventilation after reintubation. For patients who had a tracheostomy, the date of weaning off in mechanical ventilation was defined as the date of weaning off in mechanical ventilation after tracheostomy.

The secondary outcomes were reintubation, death from all causes, and improvement of diaphragm position assessed by chest radiographs. We defined reintubation as intubation within 72 h after extubation. Death and improvement of diaphragm position were observed for up to December 2019. To evaluate the long-term prognosis, the post-operative observation period was set at least 5 years. We compared chest radiographs performed before surgery and after extubation, and investigated the date of improvement of the diaphragm position to the preoperative position. Chest radiographs were performed in the supine or sitting position, while the patient was in the ICU, and basically in the standing position after leaving the ICU. Diaphragmatic elevation was defined as the location of the diaphragm within the anterior fourth rib on chest radiograph or elevation of the diaphragmatic dome apex > 1 rib interval (3 cm) compared to the preoperative level. Chest radiographs were evaluated by one physician with 10 year experience. The assessment of chest radiographs was not blinded.

### Covariates

This study was collected participants’ baseline characteristics data, including age, sex, body mass index (BMI), surgical urgency, operative time, blood loss, operative procedure, American society of anesthesiologists—physical status (ASA-PS) classification, EuroSCORE II [5, 6], Charlson comorbidity index (CCI) [7, 8], sequential organ failure assessment (SOFA) score [9] at first SBT, and history of thoracic surgery. The operative methods were differentiated into coronary artery bypass grafting (CABG), valve surgery, aortic surgery, congenital heart disease surgery, and others, and categorized as either single or combined surgery. Surgical urgency was differentiated into four levels: elective, urgent, emergency, and salvage. We set the following as potential confounders; age, sex, BMI, surgical urgency, operation time, blood loss, procedure (aortic surgery or not), and EuroSCORE II.

### Statistical analysis

The results were expressed as mean and standard deviation, median and interquartile range, or count (%). We performed survival time analysis for participants without missing covariates. The event was defined as incidence of weaning off in mechanical ventilation, death from all causes, and improvement of chest radiographs, respectively. The date of ICU admission for each participant was used as the starting point for survival time analysis.

We compared event rates by the presence of diaphragmatic dysfunction. For weaning off in mechanical ventilation and improvement of chest radiograph, we used the cumulative incidence function to calculate event rates. For the death from all causes, we used the Kaplan–Meier methods to calculate event rates.

To estimate risk-adjusted association between diaphragm dysfunction and outcomes, we used Fine-Gray models or Cox proportional hazard models. For nonfatal outcomes (incidence of weaning off in mechanical ventilation and improvement of chest radiographs), Fine-Gray models were used to account for the competing risk of death, and subdistribution hazard ratio (sHR), and 95% confidential intervals (95% CIs) were reported. For death from all causes, Cox proportional hazard models were used, and hazard ratio (HR) and 95% CIs were reported.

We calculated sHR and HR adjusted by potential confounders. Association between diaphragmatic dysfunction and reintubation was analyzed by univariate logistic regression. All analyses were conducted using Stata version 17.0 (StataCorp, Texas, USA). Statistical significance was set at *p* < 0.05 (two-tailed).

## Results

**Fig. 1:**
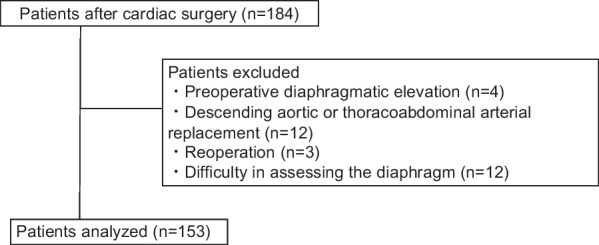
Flow chart of study

The flow chart representing this study is shown in Fig. 1; 184 patients were enrolled in this study. 31 patients were excluded. The remaining 153 patients were analyzed. Of these 153 patients analyzed, 49 patients (32.0%) had diaphragmatic dysfunction. Table 1 shows the baseline patient characteristics. The median age was 70 years (interquartile range (IQR) 65–77), 60.8% of patients were male. Elective surgery was done in 62.8% of patients, and the median operation time was 413 min (IQR 331–490). The diaphragmatic dysfunction group had longer operative time, more blood loss, more cardiopulmonary bypass cases, more aortic surgery cases, and higher EuroSCORE II than the normal group.

**Table 1:** Patient characteristics

	Total (*n* = 153)	Diaphragmatic dysfunction (*n* = 49)	Normal (*n* = 104)
Age (years)	70 (65–77)	70 (65–74)	69 (64–78)
Male	93 [60.8]	32 [66.0]	61 [58.7]
BMI, kg/m^2^	25.3 (22.8–27.0)	25.5 (22.6–26.9)	25.2 (22.8–27.0)
*Urgency*			
Elective	96 [62.8]	32 [65.3]	64 [61.5]
Urgent	36 [23.5]	9 [18.4]	27 [26.0]
Emergency	20 [13.1]	8 [16.3]	12 [11.5]
Salvage	1 [0.7]	0 [0.0]	1 [1.0]
Operation time (min)	413 (331–490)	475 (413–525)	368 (318–454)
Blood loss (mL)	2000 (1500–2890)	2606 (1971–4323)	1815 (1365–2514)
Cardiopulmonary bypass	128 [83.7]	46 [93.9]	82 [78.8]
*Operative procedure*			
CABG	61 [40.0]	11 [22.5]	50 [48.1]
Valve	32 [20.9]	12 [24.5]	20 [19.2]
Aorta	35 [22.9]	19 [38.8]	16 [15.4]
CABG + valve	13 [8.5]	6 [12.2]	7 [6.7]
CABG + aorta	1 [0.7]	0 [0.0]	1 [1.0]
Valve + aorta	7 [4.6]	1 [2.0]	6 [5.8]
Congenital heart	1 [0.7]	1 [2.0]	0 [0.0]
Other	3 [2.0]	0 [0.0]	3 [2.9]
*ASA*			
ASA1	0 [0.0]	0 [0.0]	0 [0.0]
ASA2	15 [9.8]	5 [10.2]	10 [9.6]
ASA3	119 [77.8]	38 [77.6]	81 [77.9]
ASA4,5	19 [12.4]	6 [12.2]	13 [12.5]
EuroSCORE II	2.8 (1.6–5.4)	3.4 (2.2–4.9)	2.5 (1.6–6.0)
CCI	1 (1–3)	2 (0–4)	1 (1–3)
SOFA	7 (4–9)	8 (6–9)	6 (4–8.5)
Thoracic surgery	8 [5.2]	4 [8.2]	4 [3.9]

Median (interquartile range [IQR]) or Number of persons [Percentage %], *BMI* body mass index, *CPB* cardiopulmonary bypass, *CABG* coronary artery bypass grafting, *ASA* American society of anesthesiologists, *CCI* Charlson comorbidity index, *SOFA* sequential organ failure assessment score

Table 2 shows detailed description of the diaphragmatic dysfunction group. Diaphragmatic dysfunction consisted of incomplete dysfunction in 38 patients and complete dysfunction in 11 patients. Bilateral diaphragmatic dysfunction was present in 9 patients.

**Table 2:** Details of diaphragmatic dysfunction cases

	Right (*n* = 10)	Left (*n* = 30)	Bilateral (*n* = 9)
Incomplete	9	20	6 (Right incomplete + left incomplete)
			0 (Right complete + left incomplete)
			3 (Right incomplete + left complete)
Complete	1	10	0

### Outcome

#### Primary outcome

**Fig. 2:**
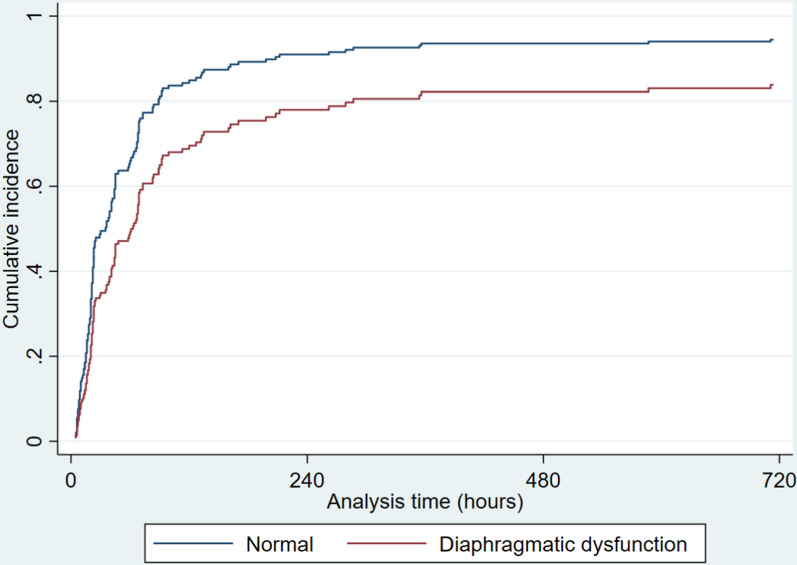
Diaphragmatic dysfunction and weaning off in mechanical ventilation

The median duration of ventilation was 23 h (IQR 15–67) and 68 h (IQR 39–114) in the normal and diaphragmatic dysfunction groups, respectively (Table 3). Figure 2 shows the cumulative incidence of weaning off in mechanical ventilation on the presence of diaphragmatic dysfunction. The longest duration of mechanical ventilation was 30 days (714 h). Except for the cases of death, all patients were weaned off in mechanical ventilation. Patients with diaphragmatic dysfunction had a longer time to wean off in mechanical ventilation compared to those without diaphragmatic dysfunction [unadjusted subdistribution HR = 0.59 (95% CIs 0.42–0.83), adjusted subdistribution HR = 0.63 (95% CIs 0.43–0.92)].

**Table 3:** Association between diaphragmatic dysfunction and weaning off in mechanical ventilation

	Duration of ventilation (hours) (IQR)	Unadjusted sHR (95% CIs)	Adjusted sHR (95% CIs)
Normal	23(15–67)	Reference	Reference
Diaphragmatic dysfunction	68(39–114)	0.59 (0.42–0.83)	0.63 (0.43–0.92)

Fine-Gray models analysis were conducted with adjustments for age, sex, BMI, surgical urgency, operative time, blood loss, procedure (aortic surgery or not), and EuroSCORE II

Median (interquartile range [IQR]), *sHR* subdistribution hazard ratio, *CIs* confidence intervals

One of the patients with diaphragmatic dysfunction was repeatedly reintubated. Later, a tracheostomy was performed, and the patient was weaned off in mechanical ventilation. Only this patient had a tracheostomy (case of complete left diaphragmatic dysfunction).

### Secondary outcome

#### Reintubation

Nine patients were reintubated during the observation period. Reintubation rate (12.2%) in the diaphragmatic dysfunction group was significantly higher than that in normal group (2.9%) (unadjusted odds ratio = 4.70, 95% CIs 1.12–19.65, *p* = 0.034, univariate logistic regression analysis) (Table 4).

**Table 4:** Association between diaphragmatic dysfunction and reintubation

	Reintubation (%)	Unadjusted Odds ratio (95% CIs)
Normal	3/104 (2.9%)	Reference
Diaphragmatic dysfunction	6/49 (12.2%)	4.70 (1.12–19.65)

*CIs* confidence intervals

### Death from all causes

**Fig. 3:**
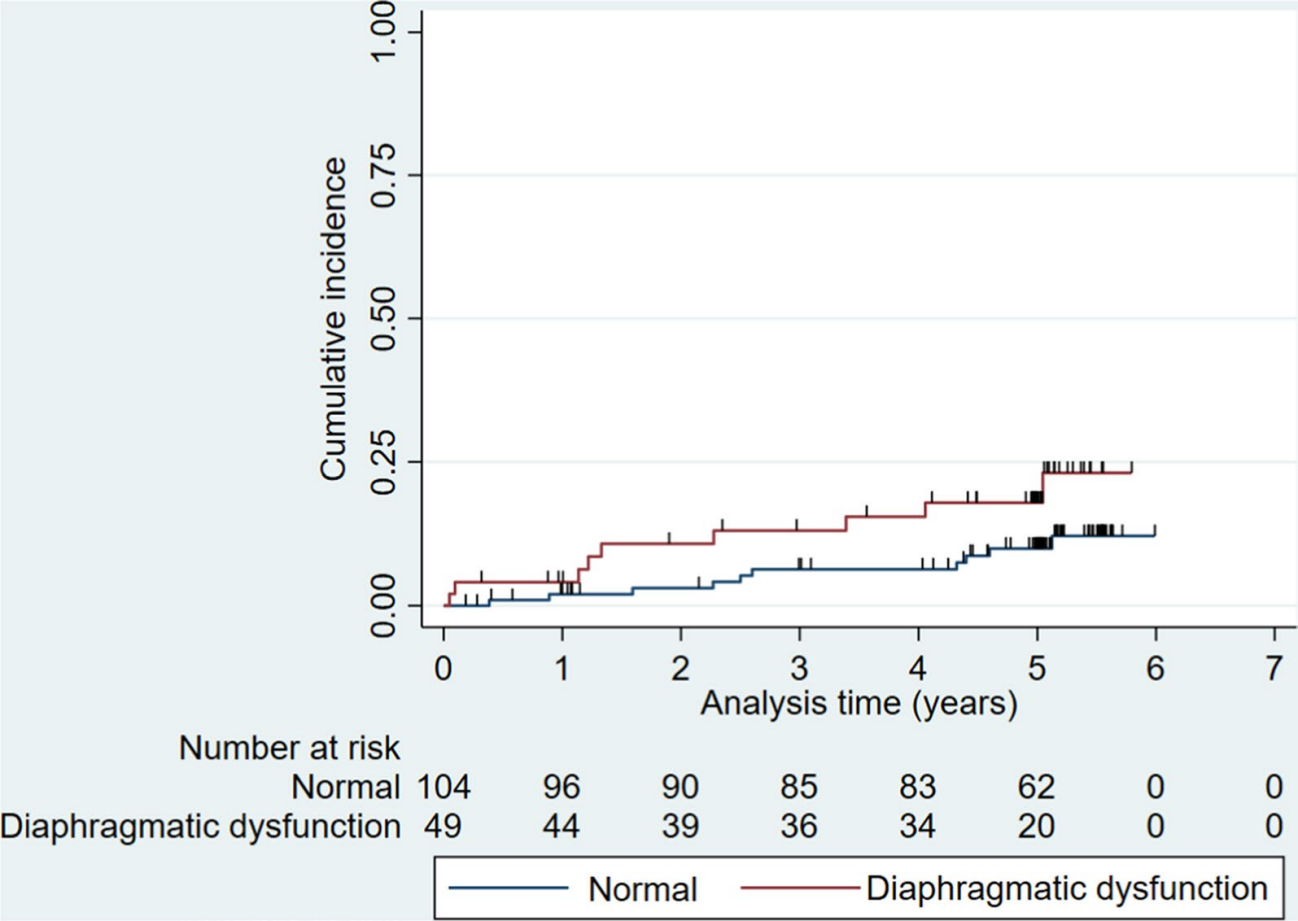
Diaphragmatic dysfunction and death from all causes

Nineteen patients died during the observation period; 4 patients died within 1 year—2 (1.9%) in the normal group and 2 (4.1%) in the diaphragmatic dysfunction group. Ten (9.6%) and 9 (18.4%) patients died during the observation period in the normal and diaphragmatic dysfunction groups, respectively. Figure 3 shows the Kaplan–Meier curves with outcome of death on the presence of diaphragmatic dysfunction. The unadjusted HR for death from all causes of diaphragmatic dysfunction was 2.14 (95% CIs 0.87–5.27), the adjusted HR was 1.64 (95% CIs 0.59–4.53) (Table 5). Although not statistically significant, HR for death from all causes tended to be higher in the diaphragmatic dysfunction group.

**Table 5:** Association between diaphragmatic dysfunction and death from all causes

	Death from all causes (%)	Unadjusted HR (95% CIs)	Adjusted HR (95% CIs)
Normal	10/104 (9.6%)	Reference	Reference
Diaphragmatic dysfunction	9/49 (18.4%)	2.14 (0.87–5.27)	1.64 (0.59–4.53)

Cox proportional hazard models analysis were conducted with adjustments for age, sex, BMI, surgical urgency, operative time, blood loss, procedure (aortic surgery or not), and EuroSCORE II

*HR* hazard ratio, *CIs* confidence intervals

### Improvement of chest radiographs

**Fig. 4:**
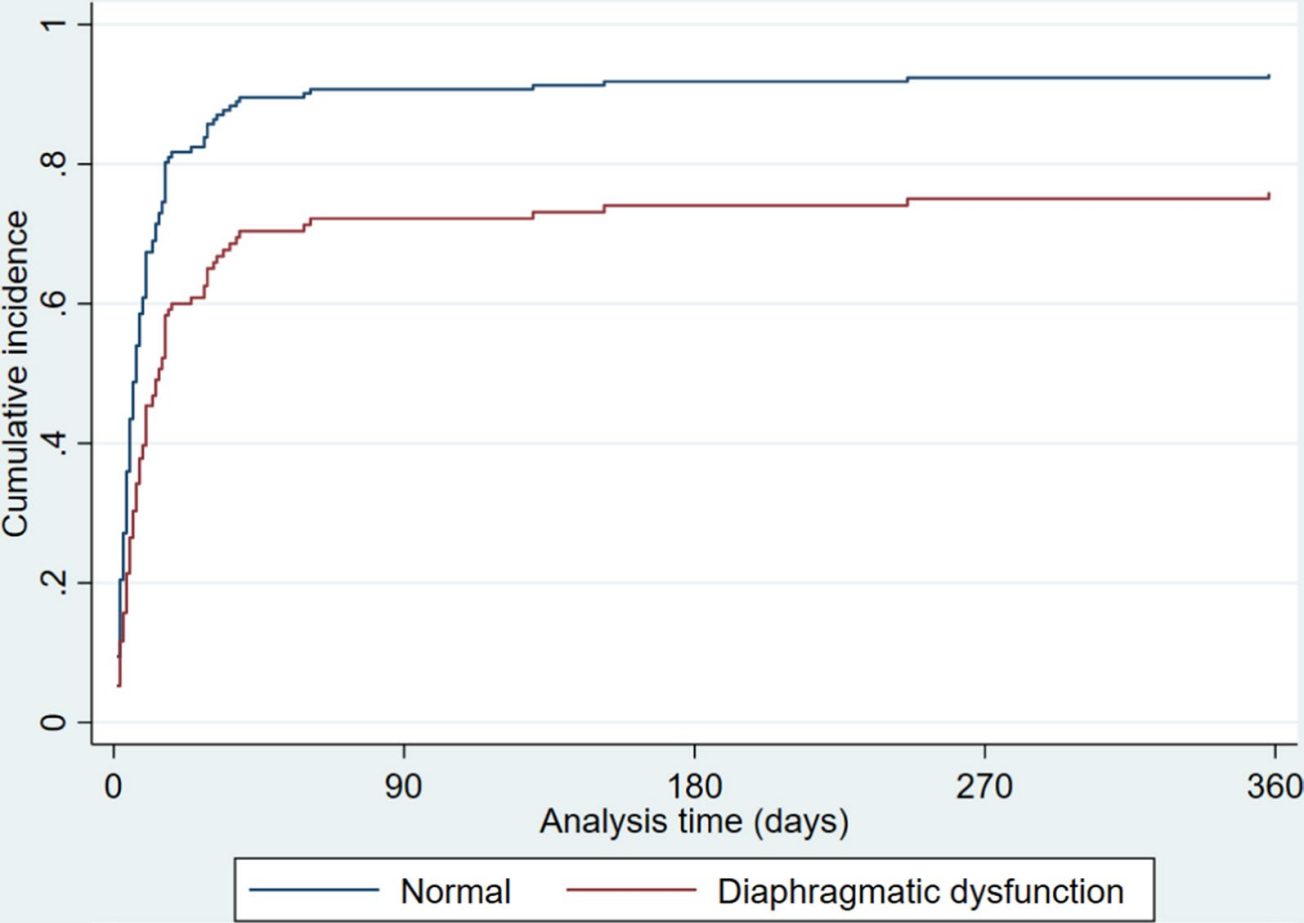
Diaphragmatic dysfunction and improvement of diaphragm position

The median time to improvement of diaphragm position on chest radiographs was 5 days (IQR 2–10) and 14 days (IQR 6–29) in the normal and diaphragmatic dysfunction groups, respectively (Table 6). Figure 4 shows the cumulative incidence of improvement of chest radiographs on the presence of diaphragmatic dysfunction. In all patients, the diaphragm position on chest X-ray improved within the observation period. The unadjusted subdistribution HR for time to improvement of chest radiograph was 0.55 (95% CIs 0.40–0.77), and the adjusted subdistribution HR was 0.54 (95% CIs 0.38–0.77). There was a statistically significant association between diaphragmatic dysfunction and time to improvement of chest radiographs. In the diaphragmatic dysfunction group, diaphragmatic elevation was found to improve, although it took a longer time.

**Table 6:** Diaphragmatic dysfunction and improvement of diaphragm position on chest X-ray

	Time to improvement (days) (IQR)	Unadjusted sHR (95% CIs)	Adjusted sHR (95% CIs)
Normal	5 (2–10)	Reference	Reference
Diaphragmatic dysfunction	14 (6–29)	0.55 (0.40–0.77)	0.54 (0.38–0.77)

Median (interquartile range [IQR]), *sHR* subdistribution hazard ratio, *CIs* confidence intervals

Fine-Gray models analysis were conducted with adjustments for age, sex, BMI, surgical urgency, operative time, blood loss, procedure (aortic surgery or not), and EuroSCORE II

## Discussion

The incidence of diaphragmatic dysfunction after adult cardiovascular surgery was 32.0% in this study. There was a significantly longer time to weaning off in mechanical ventilation, a higher rate of reintubation, and a tendency to have more mortality in the diaphragmatic dysfunction group. The time to improvement of diaphragm position on chest radiograph was significantly longer in the diaphragmatic dysfunction group.

The incidence of diaphragm dysfunction in this study was relatively higher than previous studies. In our institution, many aortic surgeries were performed and it might be related to the high incidence of diaphragmatic dysfunction. Aortic surgery is characterized by long operation time, large blood loss, and long hypothermia time with cardiopulmonary bypass. Reports of the incidence of diaphragm dysfunction after cardiovascular surgery are variable, which may be due to differences in the severity of the disease. In previous studies, post-cardiovascular diaphragmatic dysfunction in adults ranged from 18% to 36%, and post-CABG diaphragmatic dysfunction ranged from 1.31% to 60% [3, 10, 11].

Some risk factors for diaphragmatic dysfunction after cardiovascular surgery were reported in previous studies. Local hypothermia and dissection of the internal thoracic artery during CABG were reported [2]. Surgical injury or physical stretching of the diaphragmatic nerve caused by prolonged surgery, and ischemia of the diaphragm caused by bleeding were possible risk factors for diaphragmatic dysfunction. In this study, there was a trend toward more blood loss and longer operation time in the diaphragmatic dysfunction group. In addition, the diaphragmatic dysfunction group had a higher EuroSCORE II and a higher rate of aortic surgery. Few previous reports described the association between diaphragmatic dysfunction and severity scores or aortic surgery [6]. Higher EuroSCORE II and aortic surgery might be potential risk factors for diaphragmatic dysfunction. In severe cases or aortic surgery, the occurrence of postoperative diaphragmatic dysfunction should be kept in mind.

There was a statistically significant association between diaphragmatic dysfunction and duration of mechanical ventilation. The thickness of the diaphragm and intercostal muscles is reduced in mechanical ventilated patients [12]. In addition, post-cardiovascular surgery diaphragmatic dysfunction decreases respiratory function and prolongs the duration of mechanical ventilation [11, 13]; however, few studies have referred to specific durations or short- and long-term prognoses. A previous study reported no significant difference between diaphragmatic dysfunction and duration of mechanical ventilation [14]. The results were different from that in this study, because it included patients who were mechanical ventilated for more than 7 days and did not include cardiovascular surgery.

Patients who have diaphragmatic dysfunction after cardiovascular surgery may have an increased postoperative mortality. In a previous study, ICU mortality was significantly higher in patients with diaphragmatic dysfunction [14]. In this study, there was a trend toward more mortality in the diaphragmatic dysfunction group, which also supports the results of previous studies. Patients who have a diaphragmatic dysfunction after cardiovascular surgery have significantly higher reintubation rates. In a previous study, there was no reintubation rate within 72 h among 13 patients with diaphragmatic dysfunction in the ICU; however, there were two patients of reintubation after 72 h. This previous study did not show significant differences [14]. In this study, there was a significantly higher reintubation rate in the group with diaphragmatic dysfunction, which supports the results of previous studies.

The results of this study indicated that all patients in both the normal and diaphragmatic dysfunction groups were weaned off from mechanical ventilation within the observation period. These findings suggest that although the duration of the mechanical ventilation period is longer and the reintubation rate is higher in the presence of diaphragmatic dysfunction, mechanical ventilator weaning is possible if the patient is able to compensate for the decline in respiratory function. The diaphragm position on chest radiograph improved in the long term in many patients, even in the diaphragmatic dysfunction group. The degree and time needed for improvement varied from patient to patient; there were patients in which the diaphragm had improved to almost its preoperative position. Although previous studies have reported improved diaphragmatic function with reassessment of ultrasound during ICU stays [14], there were few reports on long-term diaphragmatic function. This study suggests that the diaphragmatic dysfunction after cardiovascular surgery might improve, although it would require a long time.

The strength of this study is that the functional assessment of the diaphragm was performed in all patients using ultrasonography during the spontaneous breathing test, and therefore, less data were missing. This study is useful in predicting respiratory management in patients with diaphragmatic dysfunction.

This study has several limitations. First, this was a single-center study and the results may not be applicable to other institutions. Second, the diaphragmatic function was assessed by several intensivists, and therefore, some inter- and intra-observer variations in the assessment were likely to exist. Third, due to the design of the study, it was not possible to be blinded regarding the presence or absence of diaphragmatic dysfunction, which may in fact have had an influence on the time to extubation. Fourth, the diaphragm position on chest radiograph might not precisely reflect diaphragm function [15]. Insufficient inspiration, pleural effusion, and atelectasis might affect the assessment of chest radiographs. Furthermore, the timing of chest radiograph examination was not standardized, so this study might not have accurately assessed the time to improvement of chest radiographs.

## Conclusions

Diaphragmatic dysfunction after cardiovascular surgery in adults was significantly associated with duration of mechanical ventilation and reintubation. There was a tendency to have higher death from all causes in the diaphragmatic dysfunction group.

## Data Availability

The data used and analyzed in this study are available from the corresponding author upon reasonable request.

## Abbreviations

CPB: Cardiopulmonary bypass
SBT: Spontaneous breathing trial
RSBI: Rapid shallow breathing index
NPPV: Non-invasive positive pressure ventilation
BMI: Body mass index
ASA-PS: American society of anesthesiologists—physical status
CCI: Charlson comorbidity index
SOFA: Sequential organ failure assessment
CABG: Coronary artery bypass grafting
CIs: Confidence intervals
